# Growth and Potential Damage of Human Bone-Derived Cells on Fresh and Aged Fullerene C_60_ Films

**DOI:** 10.3390/ijms14059182

**Published:** 2013-04-26

**Authors:** Ivana Kopova, Lucie Bacakova, Vasily Lavrentiev, Jiri Vacik

**Affiliations:** 1 Institute of Physiology, Academy of Sciences of the Czech Republic, Videnska 1083, 14220 Prague 4-Krc, Czech Republic; E-Mail: ivana.kopova@biomed.cas.cz; 2 Nuclear Physics Institute, Academy of Sciences of the Czech Republic, 25068 Rez near Prague, Czech Republic; E-Mails: lavrent@ujf.cas.cz (V.L.); vacik@ujf.cas.cz (J.V.)

**Keywords:** carbon nanoparticles, hydrophobicity, osteoblasts, adhesion, morphology, proliferation, cytotoxicity, gamma-H2AX, 53BP1

## Abstract

Fullerenes are nanoparticles composed of carbon atoms arranged in a spherical hollow cage-like structure. Numerous studies have evaluated the therapeutic potential of fullerene derivates against oxidative stress-associated conditions, including the prevention or treatment of arthritis. On the other hand, fullerenes are not only able to quench, but also to generate harmful reactive oxygen species. The reactivity of fullerenes may change in time due to the oxidation and polymerization of fullerenes in an air atmosphere. In this study, we therefore tested the dependence between the age of fullerene films (from one week to one year) and the proliferation, viability and metabolic activity of human osteosarcoma cells (lines MG-63 and U-2 OS). We also monitored potential membrane and DNA damage and morphological changes of the cells. After seven days of cultivation, we did not observe any cytotoxic morphological changes, such as enlarged cells or cytosolic vacuole formation. Furthermore, there was no increased level of DNA damage. The increasing age of the fullerene films did not cause enhancement of cytotoxicity. On the contrary, it resulted in an improvement in the properties of these materials, which are more suitable for cell cultivation. Therefore, fullerene films could be considered as a promising material with potential use as a bioactive coating of cell carriers for bone tissue engineering.

## 1. Introduction

Fullerenes, first discovered by Kroto *et al*. in 1985 [1], are carbon allotropes with a spherical structure consisting of more than sixty carbon atoms linked via hexagonal and pentagonal rings. Fullerene C_60_ (also termed Buckminsterfullerene or buckyball) is a remarkably stable cage-like molecule with a diameter of approximately 0.7 nm and, thus, can be defined as a nanomaterial. Thanks to their unique physicochemical properties, such as the ability to withstand high temperatures and pressures, as well as the high reactivity of these nanoparticles, fullerenes are expected to have great potential in industry as catalysts for chemical reactions, electronic equipment, additives in lubricants and fuel [2]. Due to their thirty carbon double bonds, to which free radicals can be easily added, fullerenes are considered to be radical scavengers with antioxidant properties. Therefore, they have already been utilized in cosmetics, such as facial creams and sunscreen protection products [3,4].

The structural analogy to clathrin-coated vesicles, together with the antioxidant properties of fullerenes, makes these nanoparticles highly attractive for nanomedicine, for use as drug and gene delivery agents [5–7], as well as for the prevention and treatment of specific neurodegenerative disorders caused by a hyper-production of reactive oxygen species (ROS), in such diseases as Alzheimer’s and Parkinson’s [8,9]. Furthermore, fullerenes are able to inhibit the release of allergic mediators in human mast cells and peripheral blood basophils *in vitro*, which suggests that fullerenes could control mast cell-dependent diseases, including asthma, inflammatory arthritis, heart disease and multiple sclerosis [10]. On the other hand, C_60_ is not only able to quench, but also to generate ROS after irradiation with ultraviolet or visible light. Overproduction of ROS is known to cause cell damage, such as the disintegration of plasma membrane and cleavage of DNA, which finally leads to cell death. This harmful effect of fullerenes makes them suitable for photodynamic therapies against tumors, viruses and bacteria that are resistant to multiple drugs [11–13].

Despite the promising potential of fullerenes in medicine, numerous studies have reported a toxic effect of these nanoparticles on various animals, organs, cells and microorganisms (for a review, see [14]). However, recently, it has been found that tetrahydrofuran (THF), which was widely used as a solvent of water insoluble fullerene C_60_, generates cellular toxicity. Thus, the toxic side products, such as γ-butyrolactone, 2-hydroxytetrahydrofuranol and formic acid (created during the preparation of C_60_ suspension), are probably responsible for the cytotoxicity previously attributed to fullerenes, whereas fullerenes themselves have no negative effect [15–17]. In accordance with this finding, an *in vivo* toxicity study has revealed that aqueous C_60_ suspensions prepared without using any polar organic solvent not only cause no acute or subacute toxicity in rodents, but also have a protective effect on their livers in a dose-dependent manner [18]. In addition, no irritation of skin (human volunteers) or eyes (rabbit) nor allergic risks were observed after administration of the fullerene water suspension [19]. Repeated doses of fullerenes in benzene for up to 24 weeks post-initiation also did not cause acute toxic effects, and no benign or malignant skin tumor formation was observed in mice [20]. Furthermore, inhalation experiments in rats demonstrated lower cytotoxic and proinflammatory effects of C_60_ nanoparticles in comparison with quartz particles [21] and relatively low and similar toxicity of C_60_ in both nanoparticle and microparticle forms [22].

Another promising utilization of fullerenes is the prevention and treatment of arthritis. It has been reported that in an osteoarthritis rabbit model (produced by resection of both the medial meniscus and medial collateral ligament), water soluble fullerene (purchased from Vitamin C_60_) significantly reduced articular cartilage degeneration [23]. Moreover, in adjuvant-induced arthritic rats, intra-articular treatment led to a decreasing number of osteoclasts together with reduced synovitis and alleviated bone resorption and destruction of the joints, due to the suppressive effect of C_60_ on proinflammatory cytokine production in the synovial inflammation-related cells [24,25]. Recently it has been reported that carbon nanohorns (CNH; another carbon allotrope closely related to the fullerenes), fixated on a porous polytetrafluoroethylene membrane, were used for covering the calvarial bone defect in rats. The results showed the attachment of macrophages to CNHs and acceleration of newly formed bone regeneration in the presence of these nanoparticles [26].

According to these findings mentioned in the preceding paragraph, fullerene materials appear very promising in bone tissue engineering, e.g., for surface treatment of bone implants. This idea is further supported by our earlier studies performed on fullerenes C_60_ and binary C_60_/Ti composites deposited on carbon-based or glass substrates in the form of continuous and micropatterned films [27–31]. These films provided a good support for the adhesion, growth and phenotypic maturation of human osteoblast-like MG-63 cells. The growth dynamics of these cells cultured on continuous films were similar to the dynamics on standard cell culture polystyrene dishes [29]. However, potential adverse effects of the fullerene films on cells have not been investigated in these studies. These effects could result from possible changes of physicochemical properties of the fullerene films in time, due to the oxidation and polymerization of C_60_ molecules in an air atmosphere. This could lead to changes in the reactivity of these molecules, which could influence the cytotoxicity of fullerenes. Therefore, we have decided to study the dependence between the age of C_60_ layers (from one week to one year), deposited on glass coverslips, and the adhesion, proliferation, viability and metabolic activity of human osteosarcoma cells, in order to develop a potential bioactive coating of bone implants and cell carriers applicable in bone tissue engineering. We have concentrated not only on the positive effects, but also on potential membrane and DNA damage, as well as morphological changes of cells cultivated on fullerene films.

## 2. Results and Discussion

### 2.1. Atomic Force Microscopy (AFM)

**Figure 1:**
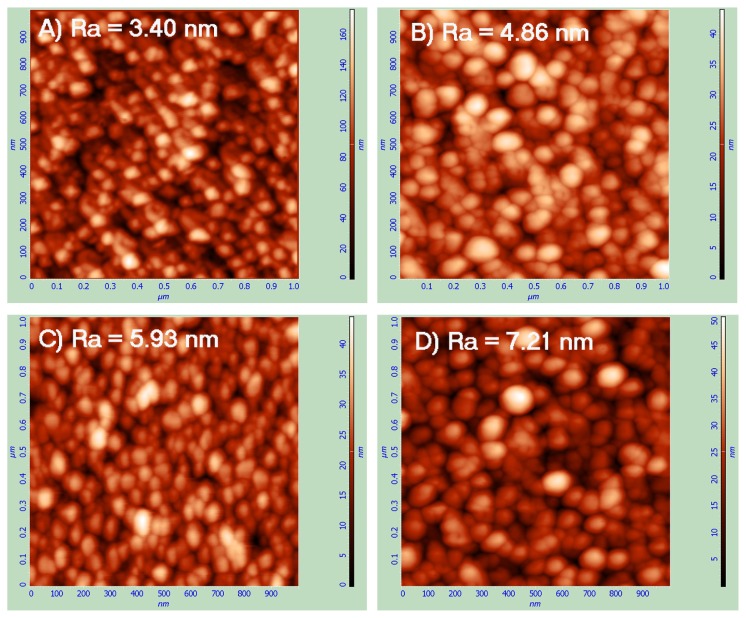
Surface morphology on the prominences (or on the continuous film; **A**,**B**) and on the grooves (**C**,**D**) of fresh (**A**,**C**) and aged (**B**,**D**) C_60_ films, evaluated by Atomic Force Microscopy (AFM).

The surface morphology and thickness of the fresh and aged C_60_ films were analyzed by Atomic Force Microscopy (AFM) (Figure 1). The AFM micrographs exhibit granular nanostructures (formed by C_60_ clusters) with a typical granule size of approximately 50 nm in the fresh films. The analysis revealed that 1 μm area roughness on the prominences, in both cases (3.40 nm on fresh and 4.86 nm on aged layers) is much smaller than the roughness on the grooves (5.93 nm on fresh and 7.12 nm on aged films). The roughness on the prominences, as well as on the grooves, is higher on the aged C_60_ layers, which could be explained by the fullerene polymerization and other changes of the C_60_ films during the aging period, leading to an increased size of the granular nanostructures (Figure 1).

**Figure 2:**
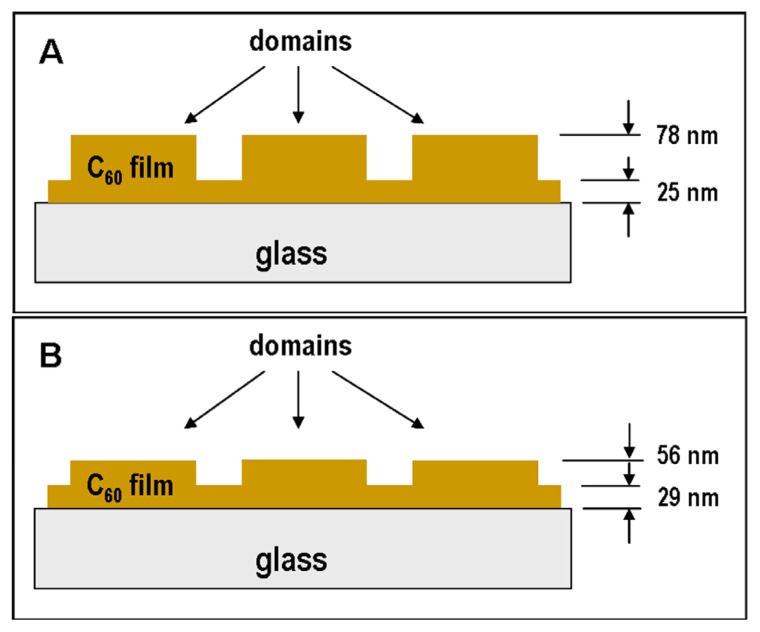
Height of the prominences and the grooves of the micropatterned fresh (**A**) and aged (**B**) C_60_ films.

It is also possible to use AFM to scan and analyze the heights of the prominences and grooves. The measurements revealed, in the case of the fresh C_60_ films, that the prominences are about twice as high as the grooves (prominences = 53 nm, grooves = 25 nm), though in the case of the aged layers, the height of the C_60_ prominences are similar to the height of the grooves (prominences = 27 nm, grooves = 29 nm; Figure 2). This decrease in height of the prominences on aged C_60_ films can be explained by post-deposition surface diffusion of the fullerene molecules in time, *i.e.*, during the aging period of fullerene films.

### 2.2. Raman Spectroscopy

The chemical composition and bonding of the fresh (*i.e.*, one week old) and aged (*i.e.*, one year old) fullerene films were characterized by several techniques, including Raman spectroscopy.

**Figure 3:**
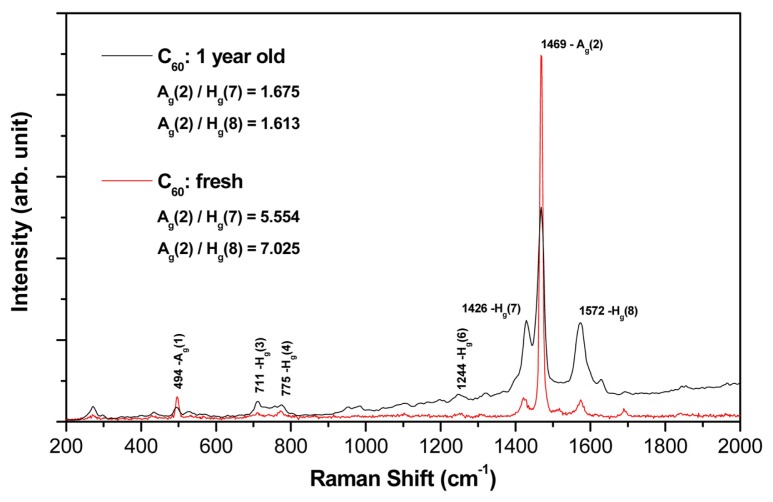
Raman spectra of the fresh and aged C_60_ films.

In Figure 3, typical micro-Raman spectra measured on both types of C_60_ films, one-year old and freshly synthesized, are depicted. In both cases, the spectra were measured on the top of the C_60_ bulges. Using the multi-peak Gaussian analysis of the H_g_(7), A_g_(2) and H_g_(8) vibration peaks, area peak ratios A_g_(2)/H_g_(7) and A_g_(2)/H_g_(8) for both films were evaluated with the following results:

$$\begin{array}{l} \text{Fresh }{\text{C}}_{60}\;\text{film}:{\text{A}}_{\text{g}}(2)/{\text{H}}_{\text{g}}(7)=5.554,{\text{A}}_{\text{g}}(2)/{\text{H}}_{\text{g}}(8)-7.205\\ \text{One}-\text{year old }{\text{C}}_{60}\;\text{film}:{\text{A}}_{\text{g}}(2)/{\text{H}}_{\text{g}}(7)=1.675,{\text{A}}_{\text{g}}(2)/{\text{H}}_{\text{g}}(8)=1.613. \end{array}$$

The Raman analysis showed:

(a) Both films (aged and fresh) exhibit typical features of the fullerene films with dominant H_g_(7), A_g_(2) and H_g_(8) peaks.
(b) In the aged samples, however, a main A_g_(2) peak (pentagonal pinch mode) dropped down dramatically (see the area peak ratios above) and showed a slight red-shift asymmetry (seen in the detailed examination of the spectrum).
(c) Interestingly, there is also a clear difference in the symmetric (A_g_) and asymmetric (H_g_) mode changes of the aged sample (in comparison with the fresh one); all symmetric mode peaks decreased in intensity, though the asymmetric modes increased.

All of these features point to the structural and bonding alterations that have developed in the aged C_60_ film—mainly due to the polymerization and partial oxidation of the fullerene molecules. The aged sample Raman spectrum also showed graphitization of the film, but this effect is rather small.

### 2.3. X-ray Photoelectron Spectroscopy (XPS)

**Figure 4:**
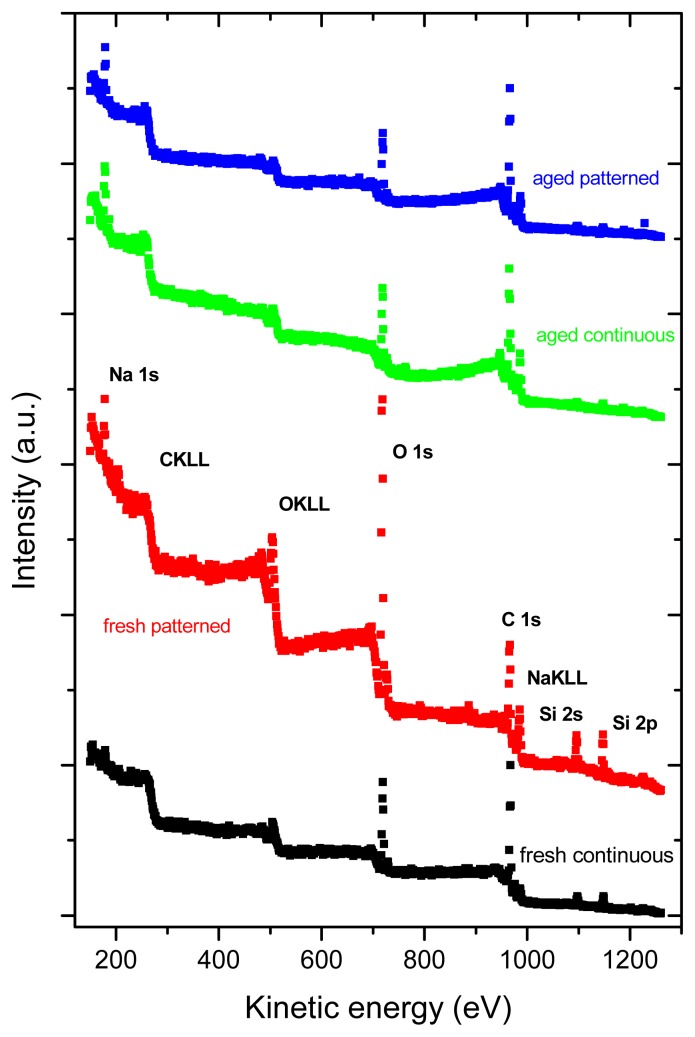
Survey XPS spectra of samples under analysis. C 1s, O 1s Si 2s, Si 2p and Na 1s are core-level photoelectron spectra from carbon, oxygen, silicon and sodium atoms. C KLL, O KLL and Na KLL are Auger transitions from carbon, oxygen and sodium atoms.

**Figure 5:**
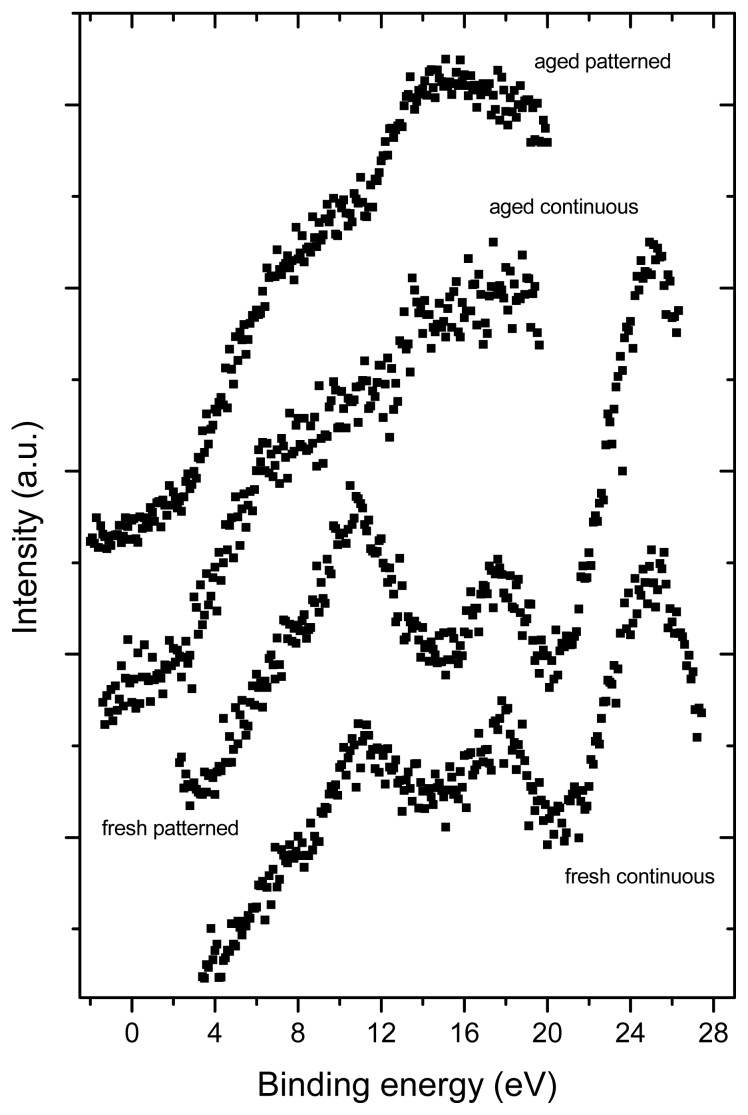
Photoemission from occupied valence bands induced by Mg Kα radiation (1253.6 eV). The spectra of fresh prepared samples show oscillations typical for fullerenes. For aged samples, the spectral features are more complex, indicating strong degradation of the fullerene molecular structure or a surface contamination by a carbon-containing species from air.

XPS revealed the presence of C, O, Si and Na in all tested samples (Figure 4). Similarly as Raman spectroscopy, XPS also showed more pronounced changes in the aged fullerene films in comparison to the fresh ones, which is indicated by a missing structure of the bands typical for fullerenes (Figure 5). This can be explained by a degradation of the fullerene molecules and/or spontaneous deposition of carbon compounds, present in the air, on the aged films. Similar carbonaceous contamination has been observed on the surface of Ti and TiNb materials developed for the construction of bone implants [27–32].

The concentration of oxygen, measured by XPS, was higher in fresh than in aged fullerene films (Tables 1 and 2). This finding might be inconsistent with the results of Raman spectroscopy, which showed that the alterations of fullerene films, such as polymerization, graphitization and partial oxidation of the fullerene molecules, were more pronounced in the aged films. However, it should be taken into account that the Raman spectroscopy measures the changes throughout the whole thickness of the films, while XPS only measures changes on the very thin surface of these films. The mean value of the inelastic mean free path of electrons is ~2–3 nm for fullerenes C_60_ and C 1s electrons of the energy of ~1000 eV). The informational depth of the method is about three times higher, *i.e.*, 6 to 9 nm. As shown by AFM, the fullerene films are much thicker, *i.e.*, from 25 to 50 nm. In addition, the presence of oxygen on the surface of the aged films could be masked by the carbonaceous contamination mentioned above.

**Figure 6:**
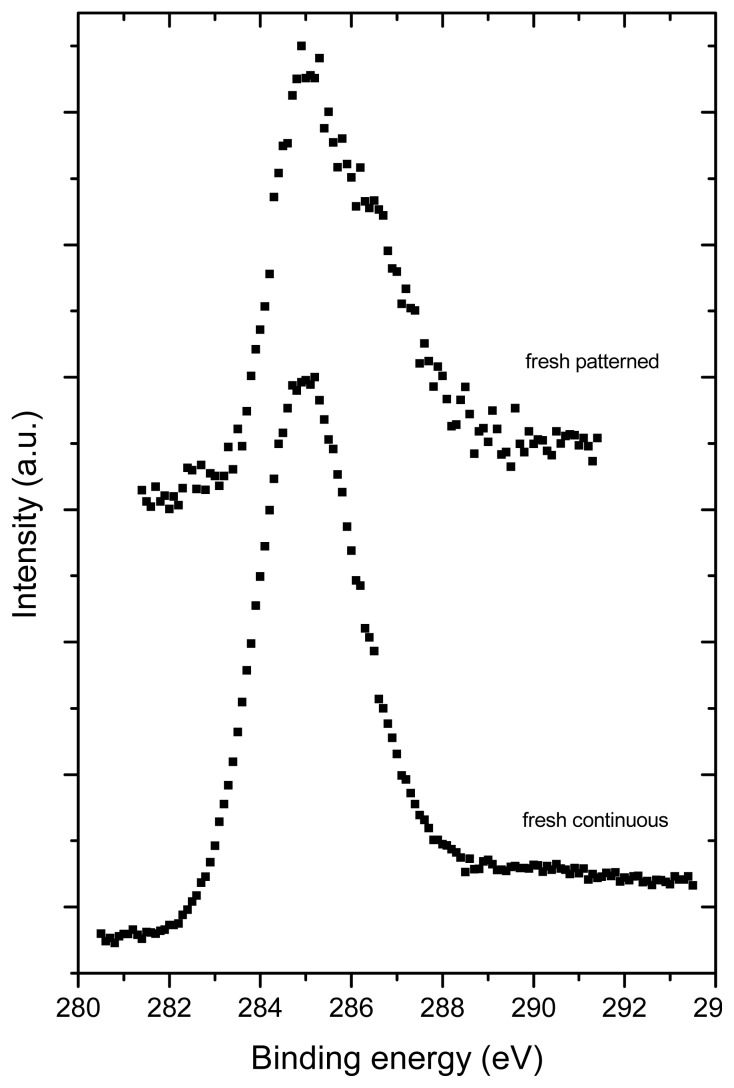
High-resolution C 1s lines recorded from fresh deposited samples. Both spectra were corrected for surface charging with respect to the Si 2p line at 103.0 eV. The shape of the bottom spectrum is similar to that of fullerene while the top spectrum is highly asymmetric due to C-O bonding states.

Interestingly, the concentration of oxygen measured by XPS was higher in fresh micropatterned films than in fresh continuous films (Figure 6, Tables 1 and 2). This can be explained by the fact that the materials with prominences and grooves have a larger surface than the flat continuous films and, thus, these surfaces can accommodate a larger number of oxygen-containing structures. As shown by AFM, on the aged materials, the height of the prominences was lower; thus, the surface of these materials became smaller and the oxygen content of both continuous and micropatterned aged surfaces equilibrated.

One should take into account also the fact that there exists competition between oxidation (fast process) and polymerization (lengthy process) and that in time, polymerization as a chemically alteration process will prevail. This may partially explain the situation why the amount of oxide is at aged samples smaller than at fresh ones.

As for the presence of Si and Na in the tested samples, angular measurements of photoemission (not shown) indicated that Si and Na are localized below the fullerene layers, *i.e.*, in the underlying glass coverslips. As the spectra were recorded from a relatively large area (specifically 1 cm^2^), these results suggest non-homogeneous coating of the glass substrates with fullerenes, *i.e.*, the absence of these films on some places or the presence of regions covered with a very thin fullerene film (~4 to 5 nm).

### 2.4. Hydrophobicity of Fullerene C_60_ Layers

**Figure 7:**
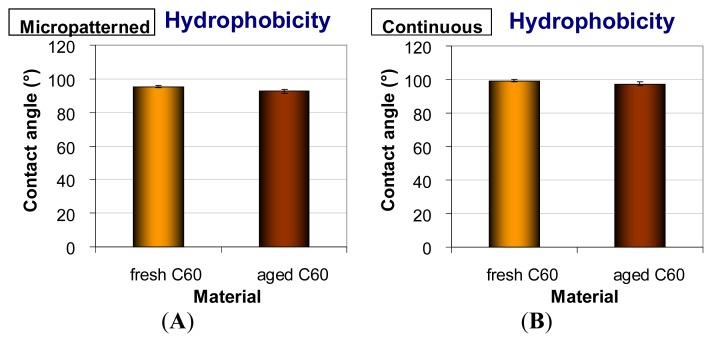
Hydrophobicity of micropatterned (**A**) or continuous (**B**) fresh and aged fullerene films.

The continuous and micropatterned layers were at a relatively high hydrophobic level. The water drop contact angles of fresh materials were 95.3° on micropatterned films and 99.3° on continuous films. Similar results were obtained in our earlier studies performed on continuous and micropatterned C_60_ films of various thicknesses [28,29]. A slight decrease of the water contact angle was observed in the aged fullerene films (92.7° on micropatterned and 97.2° on continuous films); however, this reduction was not proven to be statistically significant (Figure 7). This tendency to increase in surface wettability could be due to the changes of the fullerene films during aging, such as their polymerization, oxidation, graphitization and degradation, as revealed by the Raman spectroscopy and XPS (Figures 3 and 5). In our earlier studies, similar changes were observed on fullerene films exposed to 70% ethanol used for material sterilization ([28]; for a review, see [14]).

### 2.5. Initial Adhesion, Proliferation and Morphology of Cells on Fullerene C_60_ Layers

#### 2.5.1. Comparison of Cell Behavior on Fresh and Aged Fullerene Films

**Figure 8:**
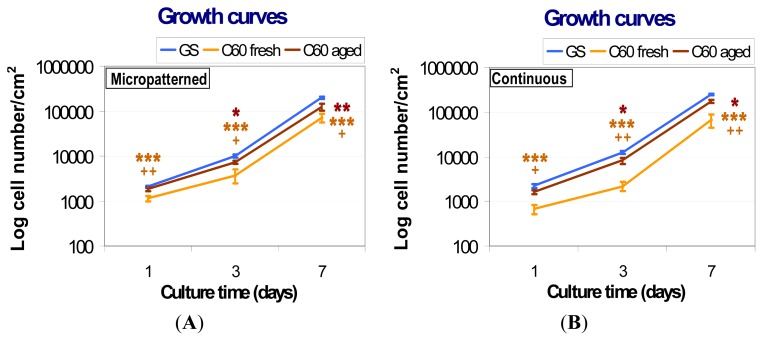
Growth curves of human osteoblast-like MG-63 cells on micropatterned (**A**) or continuous (**B**) fresh and aged fullerene films. GS, microscopic glass coverslips, reference material; * significant difference to GS; + significant difference to C_60_ aged. *p* ≤ 0.001 (***/+++); *p* ≤ 0.01 (**/++); *p* ≤ 0.05 (*/+).

**Figure 9:**
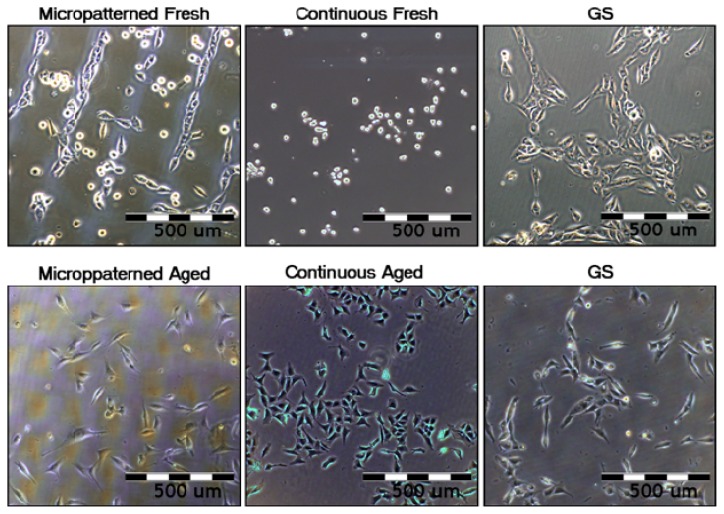
The morphology and preferential growth of human osteoblast-like MG-63 cells on micropatterned or continuous fresh and aged fullerene films on day 3 after seeding. GS, microscopic glass coverslips, reference material.

The initial adhesion of human osteoblast-like MG-63 cells seeded on fresh (*i.e.*, one week old) micropatterned and continuous fullerene films was significantly lower in comparison with the aged (*i.e.*, one year old) C_60_ layers, as well as with the reference microscopic glass coverslips (Figure 8). After three and seven days of cultivation, cells growing on both types of fresh fullerene films reached significantly lower population densities than those on the aged layers and control glass coverslips. In addition, the cells cultivated on the fresh C_60_ films were poorly spread with a rounded morphology (Figure 9).

From day one to three after seeding, the cells on fresh fullerene films proliferated more slowly than on control glass coverslips. The cell population doubling times on fresh micropatterned and continuous C_60_ films were 27.9 and 28.0 h, respectively, while on control glass coverslips, it was only 20.9 and 19.4 h. As a result of the reduced initial adhesion and growth dynamics, the cells cultured on fresh fullerene films reached significantly lower population densities on day three after seeding (Figure 8). However, after three days of cultivation, the cells on fresh fullerene films proliferated with similar growth dynamics compared to the cells on the reference material. The doubling times were 22.6 h on fresh micropatterned films, 19.5 h on fresh continuous films and 22.4 and 22.3 h on reference glass coverslips. Although the differences in growth dynamics disappeared between days three and seven, the cell population densities reached on day seven still remained significantly lower on fresh fullerene films than on glass coverslips (Figure 8). Therefore, the lower population densities on day seven can be explained by combination of a lower number of initially attached cells and reduced growth dynamics during first three days of cultivation.

Aged (*i.e.*, one year old) fullerene films provided a better support for the adhesion and growth of MG-63 cells than the fresh films. On both aged micropatterned and continuous C_60_ layers, the number of initially adhered cells was higher than on the corresponding fresh films and similar to the values found on control glass coverslips (Figure 8). Cells on both aged films were well spread, *i.e.*, of polygonal or spindle-like morphology (Figure 9).

The subsequent growth dynamics of cells cultured on aged C_60_ films were closer to those found on the control glass coverslips than in the case of fresh films. From day one to three after seeding, the doubling times on aged micropatterned and continuous C_60_ films were 24.2 and 20.6 h, respectively, and on the control glass coverslips, the values were 20.9 and 19.4 h. Between days three and seven, the doubling times on aged fullerene films (23.3 h on micropatterned and 21.9 h on continuous films) became fully comparable with the values on glass coverslips (22.4 and 22.3 h). Nevertheless, the cell population densities on days three and seven after seeding on aged fullerene films remained still lower than on the reference glass coverslips, although they were significantly higher than on fresh fullerene films (Figure 8). This can be due to the fact that even after aging, the surface hydrophobicity of C_60_ films still remained relatively high (Figure 7) when compared to the microscopic glass coverslips (Menzel Glaser, Germany) used in this study. The water drop contact angle on these coverslips cleaned with ethanol and deionized water was about 60° (data not shown here). Thus, the glass surface was moderately hydrophilic, which is considered as optimal for the cell adhesion. Similarly as nanostructured surfaces, also the moderately hydrophilic surfaces adsorb the cell adhesion-mediating molecules in an active geometrical conformation, well recognized by the cell adhesion receptors. On the contrary, on hydrophobic materials (*i.e.*, with contact angle more than 90°, which is the case of all fullerene films investigated in this study), the cell adhesion-mediating molecules are adsorbed in a rigid and denatured form, which reduces their accessibility for the cell adhesion receptors. In addition, hydrophobic surfaces preferentially adsorb albumin, which is non-adhesive for cells (for a review, see [33,34]).

The improved adhesion, morphology and proliferation of MG-63 cultured on aged C_60_ films are likely due to the changes in the fullerene films during aging, such as fragmentation, oxidation, polymerization and graphitization of fullerenes in an air atmosphere (Figures 3 and 5). These changes could lead not only to the modification of the chemical properties and the polarity of the material surface, but also to its enhanced nanoscale roughness. These factors together could result in the facilitation and enhancement of cell adhesion (for a review, see [14,33–35]). For example, substrates with nanoscale irregularities promote the adsorption of cell adhesion-mediating extracellular matrix molecules (e.g., fibronectin, vitronectin) present in the serum supplement of the culture media, in an appropriate geometrical conformation, which enables good accessibility of specific sites in these molecules (*i.e.*, RGD-containing oligopeptides) for cell adhesion receptors. In addition, these surfaces adsorb preferentially vitronectin, which is recognized mainly by osteoblasts compared to other cell types [36].

The oxygen present in the fullerene films can also play an important role in cell adhesion and growth. It has been repeatedly shown that the formation of oxygen-containing chemical functional groups on the material surface supports the cell adhesion (for a review, see [32–35]). However, on the fresh fullerene films, which contained more oxygen on their surface, the initial cell adhesion was poorer than on the aged film with a lower surface oxygen concentration. It can be supposed that on freshly deposited films, the oxygen structures (as well as the fullerenes themselves) could be present in more reactive forms harmful for cells. In accordance with this, the cell viability, measured by a trypan-blue exclusion test, was reduced on fresh fullerene films compared to the aged films and uncoated glass coverslips (see below, section 2.6). On the other hand, no DNA damage response was found in cells grown on both fresh and aged fullerene films (section 2.7).

The fresh fullerene films might be also more prone to the release of fullerene micro- and nano-particles, which can enter and damage the cells. However, in our earlier unpublished experiments, MG-63 cells were treated with fullerenes C_60_ suspended in the cell culture medium (concentration range from 0.15 μg to 30 μg/mL) for seven days. The fullerene suspensions were prepared by dissolving C_60_ in DMSO and by sonicating the suspensions for 3 h in order to prevent the formation of aggregates [37]. The results showed no reduction in proliferation after seven days long treatment with dispersed fullerenes C_60_. In addition, we did not observe any cytotoxic morphological changes, such as enlarged cells or cytosolic vacuole formation. Thus, even if the fullerenes were released from the films, most likely this release was not the main reason for the lower cell colonization of these films in comparison with control glass coverslips.

#### 2.5.2. Comparison of Cell Behavior on Micropatterned and Continuous Fullerene Films

Cells cultivated on fresh micropatterned films adhered and preferentially grew in grooves among the prominences of C_60_ (Figure 9). Similar cell behavior was also observed in our earlier studies performed on micropatterned C_60_ and hybrid C_60_/Ti films [28–31]. This has been explained by a synergistic action of certain physical and chemical properties of the fullerene bulges less appropriate for cell adhesion, such as their hydrophobicity, a relatively steep rise, as well as the tendency of spherical ball-like fullerene C_60_ molecules to diffuse out of the prominences [28]. However, on aged micropatterned C_60_ films, the preferential adhesion and growth of cells in grooves among the prominences almost disappeared (Figure 9). This could be mainly due to a decrease in the height of the prominences after diffusion of the fullerenes (Figure 2) and also due to the other changes of the fullerenes during aging, mentioned above.

Interestingly, the cell morphology and spreading was poorer on fresh continuous than on fresh micropatterned films (Figure 9). However, cytotoxic morphological changes, such as enlarged cells or cytosolic vacuole formation, were not observed on both forms of fresh C_60_ films. Thus, these differences could be explained by a different morphology of the films. In our earlier studies, the cell attachment, spreading and growth on a terpolymer of polytetrafluoroethylene, polyvinyl difluoride and polypropylene (PTFE/PVDF/PP) was markedly improved after the addition of carbon nanotubes to these polymers, which was attributed to the increased micro- and nano-scale surface roughness. At the same time, the material surface hydrophobicity was relatively high and did not differ significantly between the pure and nanotube-modified terpolymers [14,27]. Similarly, in the present study, the microscale surface roughness of the micropatterned fullerene film, hierarchically combined with a nanostructure, could compensate, at least to a certain degree, a relatively high hydrophobicity of the fullerene films.

### 2.6. Metabolic Activity and Viability of Cells on Fullerene C_60_ Layers

**Figure 10:**
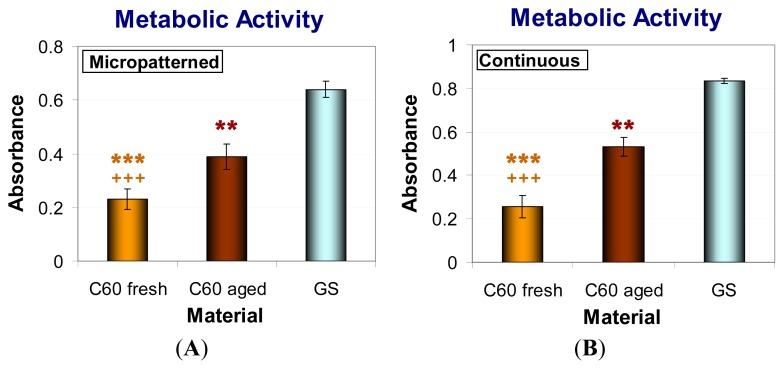
Metabolic activity measured per cultures of human osteoblast-like MG-63 cells on day seven after seeding on micropatterned (**A**) or continuous (**B**) fresh and aged fullerene films. GS, microscopic glass coverslips, reference material; * significant difference to GS; + significant difference to C_60_ aged. *p* ≤ 0.001 (***/+++); *p* ≤ 0.01 (**/++).

In order to investigate the metabolic activity of fresh and aged C_60_ films, the XTT assay, measuring the activity of mitochondrial enzymes, was performed. This activity is considered to be proportional to the cell number; therefore, this assay is often used for evaluation the cell proliferation. Thus, proportionally to the lower cell number, MG-63 cultivated for seven days on both forms of C_60_ layers (micropatterned and continuous) showed significantly reduced metabolic activity in comparison with cells grown on control glass coverslips. As in the case of population densities, the fresh materials caused a much lower metabolic activity of cells than aged C_60_ films (Figure 10). Similar results were obtained after three days of cultivation (data not shown).

**Figure 11:**
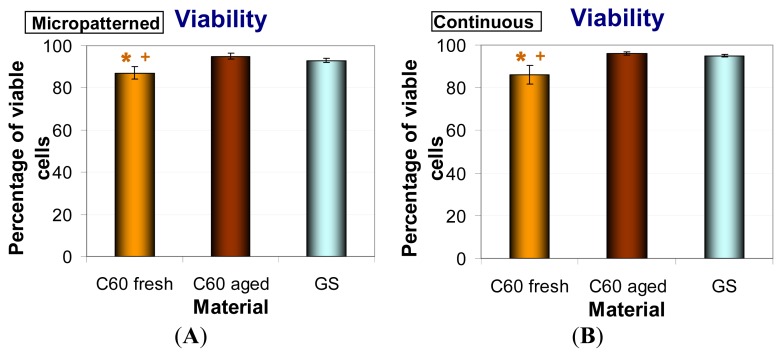
Viability of human osteoblast-like MG-63 cells after seven days of cultivation on micropatterned (**A**) or continuous (**B**) fresh and aged fullerene films. GS, microscopic glass coverslips, reference material; * significant difference to GS; + significant difference to C_60_ aged. *p* ≤ 0.05 (*/+).

Cell viability was analyzed by trypan blue staining. This dye penetrates through the damaged cell membrane and stains non-viable cells. We found that the cells growing on all tested fullerene films were highly viable (over 85%). The viability of MG-63 was reduced on both forms of fresh C_60_ layers in comparison with glass coverslips and with aged fullerene films. However, the viability of cells growing on aged C_60_ layers was comparable to that on the reference glass coverslips (Figure 11).

### 2.7. DNA Damage Response

**Figure 12:**
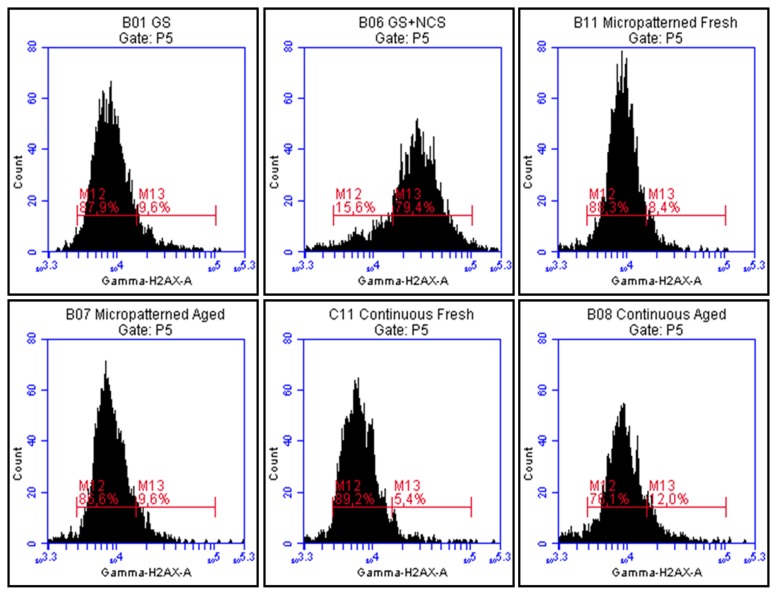
Flow cytometry of a marker of DNA damage response: gamma-H2AX in human osteoblast-like U-2 OS cells on micropatterned or continuous fresh and aged fullerene films after seven days of cultivation. GS, microscopic glass coverslips, reference material; GS + NCS, positive control to phosphorylation of histone H2AX (gamma-H2AX), induced by 1 h incubation of U-2 OS cells in neocarzinostatin (NCS; 700 ng/mL). M12 defines the percentage of cells with no increase of DNA damage (obtained from cells growing on reference material, GS); M13 defines the percentage of cells with increased DNA damage response represented by enhanced phosphorylation of histone H2AX (obtained from cells incubated with NCS).

**Figure 13:**
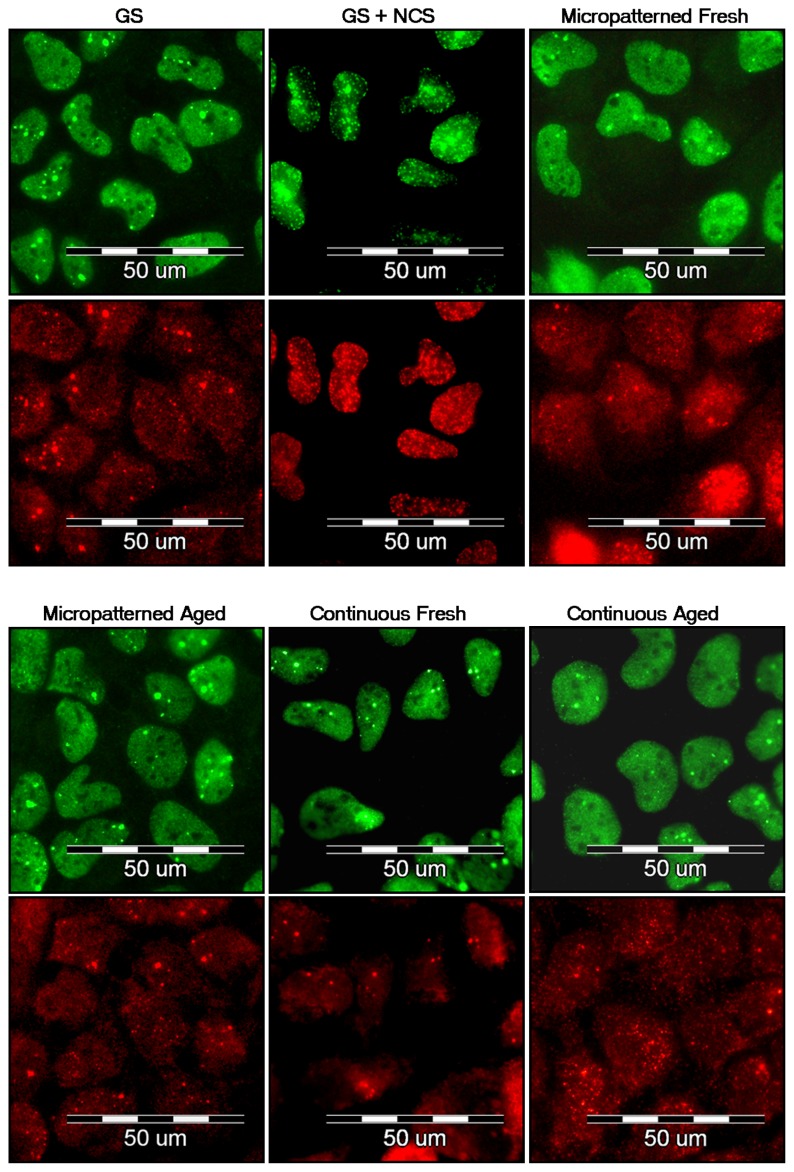
Immunofluorescence staining of markers of DNA damage response: 53BP1 (green) and gamma-H2AX (red) in human osteoblast-like U-2 OS cells on micropatterned or continuous fresh and aged fullerene films after seven days of cultivation. GS, microscopic glass coverslips, reference material; GS + NCS, positive control to DNA damage response, induced by 1 h incubation of U-2 OS cells in neocarzinostatin (NCS; 700 ng/mL).

It has been reported that fullerenes are able to bind directly to the minor and major grooves of double-strand DNA and form a stable complex, which may have a negative impact on the self-repairing process of the dsDNA, leading to the potential cytotoxic effect of fullerenes [38,39]. Therefore, we have studied the DNA damage response (DDR) of cells growing on fullerene films, by markers of DNA double strand breaks. For this purpose, the osteosarcoma cell line U-2 OS was used rather than MG-63, which is p53-deficient. Gamma-HA2X (phosphorylated histone H2AX, a marker of early DDR) and 53BP1 (p53 binding protein), whose focal recruitment depend on a number of upstream factors, were evaluated. After three and seven days of cultivation on both types of fresh and aged fullerene films, the level of gamma-H2AX phosphorylation was analyzed by flow cytometry. The results show no increase in the percentage of cells with enhanced phosphorylation of histone H2AX cultured either on fresh or aged fullerene films in comparison to the reference glass coverslips (Figure 12). Furthermore, the visualization of both DDR markers by immunofluorescence staining also revealed no increased recruitment and formation of either gamma-H2AX or 53BP1 foci (Figure 13).

## 3. Experimental Section

### 3.1. Material Deposition and Storage Condition

Thin C_60_ fullerene films were synthesized in the Molecular Beam Epitaxy (MBE) systems (in NPI ASCR Rez) by evaporation of the C_60_ phase under certain deposition kinetics: background pressure during deposition ~5 × 10^−7^ Torr; deposition rate of the C_60_ phase DR(C_60_) ~5 nm/min; temperature of the substrates during deposition ~room temperature. The fullerene layers were deposited on the selected glass coverslips (Menzel Glaser, Braunschweig, Germany; diameter 12 mm) either as continuous films or through a metallic mesh with regular rectangular openings (100 μm × 150 μm) as a micropatterned array of prominences and grooves. To vaporize the C_60_ phase material, resistive filament heating of the 99.99% pure C_60_ powder was used. The samples were stored in air atmosphere at room temperature in a dark and dry place and evaluated either one week after deposition (fresh samples) or after one year (aged samples).

### 3.2. Atomic Force Microscopy (AFM)

Surface morphology and thickness of the C_60_ films were analyzed by an Atomic Force Microscopy (AFM microscope NTEGRA, NT-MDT) using a static (contact) mode. The scanning area was selected either as 1000 nm or 100 μm (the presented micrographs, see Figure 1A,B show only 1000 nm scans).

### 3.3. Raman Spectroscopy

For analysis of the C_60_ films, a Renishaw 2000 imaging microscope (using the 514 nm Ar laser) was applied. The measurements were performed using the low laser power, *i.e.*, (<1 mW) in order to avoid fragmentation of the C_60_ molecules. The spectra were measured on the top of the C_60_ prominences, using the multi-peak Gaussian analysis of the H_g_(7), A_g_(2) and H_g_(8) vibration peaks. Area peak ratios A_g_(2)/H_g_(7) and A_g_(2)/H_g_(8) were evaluated.

### 3.4. X-ray Photoelectron Microscopy (XPS)

The XPS photoelectron spectra were recorded using an angle-resolved photoelectron spectrometer ADES 400 (VG Scientific, East Grinstead, England) operating at a base pressure of 1 × 10^−10^ Torr. The system is equipped with an X-ray excitation source and a rotatable hemispherical electron energy analyzer. The spectra were recorded using Mg Kα radiation with the pass energy of 100 eV and 20 eV; the incidence angle was 70° with respect to the sample surface normal and the emission angle along the surface normal. The overall energy resolution was 1.2 eV. An area of 1 × 1 cm on the material surface was exposed to X-rays; thus, the spectra represented a mean value of the signal from this area.

The surface composition of the materials was determined from photoelectron peak areas after Shirley’s inelastic background subtraction. Assuming a simple model of a semi-infinite solid of homogeneous composition, the peak areas were corrected for the photoelectric cross-sections [40], electron inelastic mean free paths [41] and transmission function of the spectrometer used [42]. Experimental uncertainties accompanied with XPS quantitative analysis, assessed on separate experiments with several standard materials, were estimated to be below 7%. The value covers overall uncertainties of the method that are mostly introduced by the background subtraction and the procedure used for the calculation of concentrations from intensities of spectral lines. High-energy resolution C 1s photoelectron spectra were recorded at the pass energy of 20 eV.

### 3.5. Measurement of Wettability

The surface wettability of the fullerene films was estimated from the contact angle measured by a material-water droplet system using a reflection goniometer (SEE System, Masaryk University, Brno, Czech Republic). Data were presented as the mean ± standard error of the mean (SEM) obtained from 10 measurements.

### 3.6. Cells and Culture Conditions

Since the samples were prepared under aseptic conditions (assured by the high temperature), sterilization was not performed in order to avoid potential damage to the fullerene molecules by irradiation, heating or chemicals. The fullerene-coated glass coverslips were inserted into polystyrene 24-well tissue culture plates (TPP, Trasadingen, Switzerland; diameter 15.4 mm and growth surface 1.862 cm^2^, according to the manufacturer’s data) and repeatedly rinsed in phosphate-buffered saline (PBS; Sigma, St. Louis, MO, USA). The samples were seeded with human osteosarcoma cell lines MG-63 (European Collection of Cell Cultures, Salisbury, England) in the initial density of 5370 cells/cm^2^ (10,000 cells per well) or U-2 OS cell line (ATCC-LGC, Cat. No. HTB-96; Manassas, VA, USA) in densities ranging from 4300 cells/cm^2^ (8000 cells per well) to 16,100 cells/cm^2^ (30,000 cells per well). Both cell lines were cultured for 7 days in 1 mL of Dulbecco’s Modified Eagle’s Medium (Sigma, St. Louis, MO, USA, Cat. No. D5648), supplemented with 10% fetal bovine serum (Sebak GmbH, Ingelheim, Germany) and gentamicin (40 μg/mL; LEK, Ljubljana, Slovenia) at 37 °C in a humidified air atmosphere containing 5% of CO_2_. Uncoated microscopic glass coverslips (Menzel Glaser, Braunschweig, Germany; diameter 12 mm) were used as reference material. Data from three separate experiments were evaluated. For each experimental group and time interval, three samples were analyzed.

### 3.7. Evaluation of Cell Morphology, Initial Adhesion and Proliferation (Growth Curves)

MG-63 cells were cultured for 7 days (seeding density 5370 cells/cm^2^; 10,000 cells per well). The evaluation of cell morphology was performed on days 1, 3 and 7 after seeding by using an IX-71 microscope equipped with a DP-71 digital camera (Olympus, Shinjuku, Tokyo, Japan). Immediately after that, each sample was transferred to fresh polystyrene 24-well tissue culture plates and rinsed with PBS. The cells were detached by a trypsin-EDTA solution (Sigma, St. Louis, MO, USA, Cat. No. T4174) and counted using a Bürker haemocytometer (days 1 and 3) or Vi-Cell XR analyzer on day 7 (Beckman Coulter, Fullerton, CA, USA). The obtained cell numbers were expressed as cell population densities/cm^2^ and used for the construction of growth curves and calculation of the cell population doubling time according the following formula:

$$DT=\text{log}\;2\frac{t-t_{0}}{\text{log}\;N_{t}-\text{log}\;N_{t_{0}}}$$

where *t*_0_ and *t* represent earlier and later time intervals after seeding, respectively, and *N*_t0_ and *N*_t_ the number of cells at these intervals.

Data from three separate experiments were analyzed. For each experimental group and time interval, three parallel samples were evaluated.

### 3.8. Evaluation of Cell Metabolic Activity

In order to investigate the metabolic activity of cells (which is an indirect measure of the cell proliferation activity), the commercial Cell Proliferation Kit II XTT (Roche, Basel, Switzerland, Cat. No. 11 465 015 001) was used. This is a colorimetric assays based on the cleavage of the yellow tetrazolium salt XTT (2,3-bis(2-methoxy-4-nitro-5-sulphophenyl)-2*H*-tetrazolium-5-carboxanilide) to a soluble orange formazane derivate by mitochondrial enzymes from metabolically active cells. The formazane dye is directly quantified by a spectrophotometer. After 3 and 7 days of cultivation, all samples were transferred to fresh polystyrene 24-well tissue culture plates and rinsed with PBS. To each sample, 1 mL solution of XTT and Dulbecco’s Modified Eagle’s Medium without Phenol Red (Gibco, Cat. No 11053-028) supplemented with 10% fetal bovine serum (Sebak GmbH, Ingelheim, Germany) and gentamicin (40 μg/mL; LEK, Ljubljana, Slovenia) in the ratio of 1 XTT to 2 DMEM was added (according the manufacturer’s protocol). After 4–6 h of incubation at 37 °C in a humidified air atmosphere containing 5% of CO_2_, absorbance of the resulting solution was measured at a wavelength of 470 nm against the reference value of 650 nm.

As the blank samples, a solution from C_60_ coated, as well as uncoated microscopic glass coverslips without seeded cells was used. Data from three separate experiments were analyzed. For each experimental group and time interval, three parallel samples were used and the solution from each well was divided into 8 parallel wells.

### 3.9. Evaluation of Membrane Damage and Cell Viability

On day 7 after seeding, cell viability and membrane damage of the cells were detected by trypan blue staining performed during cell counting in the Vi-Cell XR analyzer (Beckman Coulter, Fullerton, CA, USA). Data from three separate experiments were analyzed. For each experimental group 50 images from three parallel samples were evaluated.

### 3.10. Evaluation of DNA Damage Response

In order to investigate potential DNA damage of cells, osteosarcoma cell line U-2 OS was used instead of MG-63, which is p53 deficient. After 3 and 7 days of cultivation, DNA damage response was evaluated by immunofluorescence staining analyzed by fluorescence microscopy and flow cytometry.

The samples for microscopy were rinsed with PBS and fixed with 4% paraformaldehyde (PFA; Sigma, St. Louis, MO, USA) for 20 min at room temperature. Subsequently, the cells were permeabilized with 0.1% Triton X-100 in PBS (Sigma, St. Louis, MO, USA) for 20 min at room temperature. This solution also contained 1% bovine serum albumin for blocking non-specific binding sites for antibodies. The samples were incubated with primary antibodies anti-53BP1 (0.2 μg/mL; Santa Cruz Biotech, Dallas, TX, USA; clone H-300) and anti-H2A.X-Phosphorylated Ser139 (0.4 μg/mL; Millipore, Billerica, MA, USA; clone JBW301) for 1 h, followed by secondary antibodies coupled to Alexa Fluor 488 and 546 (4 μg/mL; Invitrogen, Molecular Probes, Eugene, OR, USA) for 1 h. Cells were then mounted with microscopic glass coverslips using a Gel/Mount permanent fluorescence-preserving aqueous mounting medium (Biomeda Corporation, Foster City, CA, USA) and evaluated under the epifluorescence microscope IX-71 (Olympus, Shinjuku, Tokyo, Japan) equipped with the digital camera DP-71 (Olympus, Shinjuku, Tokyo, Japan).

The samples analyzed by flow cytometry were prepared using the same protocol as those for microscopy, except that all steps were performed in suspension, not on microscopic glass coverslips. After 3 and 7 days of cultivation, three parallel samples from each experimental group were transferred to new polystyrene 24-well tissue culture plates and rinsed with PBS. The cells were detached by a trypsin-EDTA solution. The suspensions of three parallel samples were mixed together into one tube. For flow cytometry, an Alexa Fluor 488 anti-H2A.X-Phosphorylated (Ser139) antibody (5 μg/L million cells; BioLegend, San Diego, CA, USA; clone 2F3) was used. After 1 h of incubation with antibody, cells were rinsed and resuspended in PBS. The samples were analyzed by Accuri C6 Flow Cytometer (BD Biosciences, Franklin Lakes, NJ, USA).

U-2 OS treated with neocarzinostatin (NCS; 700 ng/mL; Sigma, St. Louis, MO, USA) for 1 h were used as a positive control for markers of DNA damage response. The cells were fixed 3 h after treatment with NCS. In order to confirm the results, the immunofluorescence staining, as well as flow cytometry analysis was repeated twice.

### 3.11. Statistical Analysis

Data were presented as the mean ± SEM (Standard Error of the Mean) obtained from three separate experiments. Three samples for each experimental group and time interval were evaluated. A comparison between three groups was analyzed with the ANOVA, Student–Newman–Keuls Method. In the case of two groups, the Student’s *t*-test for unpaired data was used. *p*-values less than 0.05 were considered statistically significant.

## 4. Conclusions

Our study revealed that the colonization of fullerene C_60_ films with human osteoblast-like MG 63 cells was lower in comparison with control microscopic glass coverslips, which served as substrates for the fullerene deposition. This was indicated by lower cell numbers and lower metabolic activity, measured by XTT test, of cells on the C_60_ films. On C_60_ films with micropatterned morphology, the cells adhered preferentially in grooves among the prominences. All these differences were more pronounced on fresh (*i.e.*, one week old) than on aged (*i.e.*, one year old) fullerene films. On the fresh films, also the cell viability, measured by a trypan blue exclusion test, was lower than on control glass coverslips and aged fullerene layers. Nevertheless, studies performed on human osteoblast-like U-2 OS cells revealed no DNA damage response of these cells cultivated on fresh or aged fullerene films. The increasing age of the fullerene films resulted in an improvement of the physicochemical properties of these materials, which became more suitable for cell cultivation. Therefore, fullerene films could be considered as promising materials in bone tissue engineering, namely for potential coating of bone implants.

## Figures and Tables

**Table 1:** Quantitative analysis of the surface of the tested samples.

Sample	C	O	Si	Na	F
		
	(at.%)	(at.%)	(at.%)	(at.%)	(at.%)
Fresh, continuous	57.2	29.3	11.3	2.3	-
Fresh, micropatterned	43.8	36.1	16.4	3.6	-
Aged, continuous	72.2	16.4	3.8	7.6	-
Aged, micropatterned	73.7	15.6	4.9	5.8	-
Glass substrate	21.9	49.4	22.4	4.1	2.3

**Table 2:** Correction of the data from Table 1 performed on an assumption that Si and Na originate from the glass substrate and are in the oxide state (*i.e.*, SiO_2_, Na_2_O).

Sample	C (at.%)	O (at.%)
Fresh, continuous	91	9.0
Fresh, micropatterned	80.0	20.0
Aged, continuous	93.5	6.5
Aged, micropatterned	96.2	3.8
Fullerene powder	95.2	4.8

## References

[1] Kroto H.W., Heath J.R., Obrien S.C., Curl R.F., Smalley R.E. (1985). C60: Buckminsterfullerene. Nature.

[2] Aitken R.J., Chaudhry M.Q., Boxall A.B.A., Hull M. (2006). Manufacture and use of nanomaterials: Current status in the UK and global trends. Occup. Med.

[3] Kato S., Taira H., Aoshima H., Saitoh Y., Miwa N. (2010). Clinical evaluation of fullerene-C60 dissolved in squalane for anti-wrinkle cosmetics. J. Nanosci. Nanotechnol.

[4] Kato S., Aoshima H., Saitoh Y., Miwa N. (2010). Fullerene-C60/liposome complex: Defensive effects against UVA-induced damages in skin structure, nucleus and collagen type I/IV fibrils, and the permeability into human skin tissue. J. Photochem. Photobiol. B Biol.

[5] Foley S., Crowley C., Smaihi M., Bonfils C., Erlanger B.F., Seta P., Larroque C. (2002). Cellular localisation of a water-soluble fullerene derivative. Biochem. Biophys. Res. Commun.

[6] Venkatesan N., Yoshimitsu J., Ito Y., Shibata N., Takada K. (2005). Liquid filled nanoparticles as a drug delivery tool for protein therapeutics. Biomaterials.

[7] Isobe H., Nakanishi W., Tomita N., Jinno S., Okayama H., Nakamura E. (2006). Nonviral gene delivery by tetraamino fullerene. Mol. Pharm.

[8] Dugan L.L., Gabrielsen J.K., Yu S.P., Lin T.S., Choi D.W. (1996). Buckminsterfullerenol free radical scavengers reduce excitotoxic and apoptotic death of cultured cortical neurons. Neurobiol. Dis.

[9] Dugan L.L., Lovett E.G., Quick K.L., Lotharius J., Lin T.T., O’Malley K.L. (2001). Fullerene-based antioxidants and neurodegenerative disorders. Parkinsonism Related Disord.

[10] Ryan J.J., Bateman H.R., Stover A., Gomez G., Norton S.K., Zhao W., Schwartz L.B., Lenk R., Kepley C.L. (2007). Fullerene nanomaterials inhibit the allergic response. J. Immunol.

[11] Tabata Y., Murakami Y., Ikada Y. (1997). Photodynamic effect of polyethylene glycol-modified fullerene on tumor. Jpn. J. Cancer Res.

[12] Tegos G.P., Demidova T.N., Arcila-Lopez D., Lee H., Wharton T., Gali H., Hamblin M.R. (2005). Cationic fullerenes are effective and selective antimicrobial photosensitizers. Chem. Biol.

[13] Käsermann F., Kempf C. (1997). Photodynamic inactivation of enveloped viruses by buckminsterfullerene. Antivir. Res.

[14] Bacakova L., Grausova L., Vandrovcova M., Vacik J., Frazcek A., Blazewicz S., Kromka A., Rezek B., Vanecek M., Nesladek M., Lombardi S.L. (2008). Carbon Nanoparticles as Substrates for Cell Adhesion and Growth. Nanoparticles: New Research.

[15] Spohn P., Hirsch C., Hasler F., Bruinink A., Krug H.F., Wick P. (2009). C60 fullerene: A powerful antioxidant or a damaging agent? The importance of an in-depth material characterization prior to toxicity assays. Environ. Pollut.

[16] Kovochich M., Espinasse B., Auffan M., Hotze E.M., Wessel L., Xia T., Nel A.E., Wiesner M.R. (2009). Comparative toxicity of C60 aggregates toward mammalian cells: Role of tetrahydrofuran (THF) decomposition. Environ. Sci. Technol.

[17] Henry T.B., Menn F.-M., Fleming J.T., Wilgus J., Compton R.N., Sayler G.S. (2007). Attributing effects of aqueous C60 nano-aggregates to tetrahydrofuran decomposition products in larval zebrafish by assessment of gene expression. Environ. Health Perspect.

[18] Gharbi N., Pressac M., Hadchouel M., Szwarc H., Wilson S.R., Moussa F. (2005). [60]fullerene is a powerful antioxidant *in vivo* with no acute or subacute toxicity. Nano Lett.

[19] Huczko A., Lange H., Calco E. (1999). Fullerenes: Experimental evidence for a null risk of skin irritation and allergy. Full. Sci. Technol.

[20] Nelson M.A., Domann F.E., Bowden G.T., Hooser S.B., Fernando Q., Carter D.E. (1993). Effects of acute and subchronic exposure of topically applied fullerene extracts on the mouse skin. Toxicol. Ind. Health.

[21] Sayes C.M., Marchione A.A., Reed K.L., Warheit D.B. (2007). Comparative pulmonary toxicity assessments of C60 water suspensions in rats: Few differences in fullerene toxicity *in vivo* in contrast to *in vitro* profiles. Nano Lett.

[22] Baker G.L., Gupta A., Clark M.L., Valenzuela B.R., Staska L.M., Harbo S.J., Pierce J.T., Dill J.A. (2008). Inhalation toxicity and lung toxicokinetics of C60 fullerene nanoparticles and microparticles. Toxicol. Sci.

[23] Yudoh K., Shishido K., Murayama H., Yano M., Matsubayashi K., Takada H., Nakamura H., Masuko K., Kato T., Nishioka K. (2007). Water-soluble C60 fullerene prevents degeneration of articular cartilage in osteoarthritis via down-regulation of chondrocyte catabolic activity and inhibition of cartilage degeneration during disease development. Arthrit. Rheum.

[24] Yudoh K., Karasawa R., Masuko K., Kato T. (2009). Water-soluble fullerene (C60) inhibits the development of arthritis in the rat model of arthritis. Int. J. Nanomed.

[25] Yudoh K., Karasawa R., Masuko K., Kato T. (2009). Water-soluble fullerene (C60) inhibits the osteoclast differentiation and bone destruction in arthritis. Int. J. Nanomed.

[26] Kasai T., Matsumura S., Iizuka T., Shiba K., Kanamori T., Yudasaka M., Iijima S., Yokoyama A. (2011). Carbon nanohorns accelerate bone regeneration in rat calvarial bone defect. Nanotechnology.

[27] Bacakova L., Grausova L., Vacik J., Fraczek A., Blazewicz S., Kromka A., Vanecek M., Svorcik V. (2007). Improved adhesion and growth of human osteoblast-like MG 63 cells on biomaterials modified with carbon nanoparticles. Diam. Related Mater.

[28] Grausova L., Vacik J., Bilkova P., Vorlicek V., Svorcik V., Soukup D., Bacakova M., Lisa V., Bacakova L. (2008). Regionally-selective adhesion and growth of human osteoblast-like MG 63 cells on micropatterned fullerene C60 layers. J. Optoelectron. Adv. Mater.

[29] Grausova L., Vacik J., Vorlicek V., Svorcik V., Slepicka P., Bilkova P., Vandrovcova M., Lisa V., Bacakova L. (2009). Fullerene C60 films of continuous and micropatterned morphology as substrates for adhesion and growth of bone cells. Diam. Related Mater.

[30] Vandrovcova M., Vacik J., Svorcik V., Slepicka P., Kasalkova N., Vorlicek V., Lavrentiev V., Vosecek V., Grausova L., Lisa V. (2008). Fullerene C60 and hybrid C60/Ti films as substrates for adhesion and growth of bone cells. Phys. Status Solidi A.

[31] Vacik J., Lavrentiev V., Novotna K., Bacakova L., Lisa V., Vorlicek V., Fajgar R. (2010). Fullerene (C60)–transitional metal (Ti) composites: Structural and biological properties of the thin films. Diam. Related Mater.

[32] Jirka I., Vandrovcova M., Frank O., Tolde Z., Plsek J., Luxbacher T., Bacakova L., Stary V. (2013). On the role of Nb-related sites of an oxidized β-TiNb alloy surface in its interaction with osteoblast-like MG-63 cells. Mater. Sci. Eng. C.

[33] Bacakova L., Svorcik V., Kimura D. (2008). Cell Colonization Control by Physical and Chemical Modification of Materials. Cell Growth Processes: New Research.

[34] Bacakova L., Filova E., Parizek M., Ruml T., Svorcik V. (2011). Modulation of cell adhesion, proliferation and differentiation on materials designed for body implants. Biotechnol. Adv.

[35] Bacakova L., Grausova L., Vacik J., Kromka A., Biederman H., Choukourov A., Stary V., Reddy B. (2011). Nanocomposite and Nanostructured Carbon-based Films as Growth Substrates for Bone Cells. Advances in Diverse Industrial Applications of Nanocomposites.

[36] Webster T.J., Ergun C., Doremus R.H., Siegel R.W., Bizios R. (2000). Specific proteins mediate enhanced osteoblast adhesion on nanophase ceramics. J. Biomed. Mater. Res.

[37] Kim K.T., Jang M.H., Kim J.Y., Kim S.D. (2010). Effect of preparation methods on toxicity of fullerene water suspensions to Japanese medaka embryos. Sci. Total Environ.

[38] Zhao X., Striolo A., Cummings P.T. (2005). C60 binds to and deforms nucleotides. Biophys. J.

[39] Xu X., Wang X., Li Y., Wang Y., Yang L. (2012). A large-scale association study for nanoparticle C60 uncovers mechanisms of nanotoxicity disrupting the native conformations of DNA/RNA. Nucleic Acids Res.

[40] Band I.M., Kharitonov Yu I., Trzhaskovskaya M.B. (1979). Photoionization cross sections and photoelectron angular distributions for X-ray line energies in the range 0.132–4.509 keV targets: 1 ≤ Z ≤ 100. At. Data Nuclear Data Tables.

[41] Tanuma S., Powell C.J., Penn D.R. (2011). Calculations of electron inelastic mean free paths. IX. Data for 41 elemental solids over the 50 eV to 30 keV range. Surf. Interface Anal.

[42] Jiricek P. (1994). Measurement of the transmission function of the hemispherical energy analyzer of ADES 400 electron spectrometer. Czechoslov. J. Phys.

